# Mohs micrographic surgery with magnet suspension of implantable cardioverter defibrillators

**DOI:** 10.1002/ski2.331

**Published:** 2024-01-06

**Authors:** Danae Trokoudes, Binoob Gopinath, Saqib Bashir

**Affiliations:** ^1^ Department of Dermatology King's College Hospital NHS Foundation Trust London UK; ^2^ King's College Hospital NHS Foundation Trust London UK

## CONFLICT OF INTEREST STATEMENT

None to declare.

## AUTHOR CONTRIBUTION

**Danae Trokoudes**: Conceptualization (equal); Formal analysis (equal). **Binoob Gopinath**: Resources (supporting). **Saqib Bashir**: Conceptualization (equal).

## FUNDING INFORMATION

This research received no specific grant from any funding agency in the public, commercial, or not‐for‐profit sectors.

## ETHICS STATEMENT

Not applicable.

## Dear Editor,

Dermatological surgeons are increasingly operating on patients with Implantable Cardioverter Defibrillators (ICDs), the management of which can vary widely. Older literature has raised concerns about Electromagnetic Interference (EMI), however more recent publications have demonstrated a low risk to the patient.^1^, ^2^ We present data suggesting a safe and practical method of managing these patients, using magnets, based on recent publications and guidelines.

A retrospective cohort study was performed to evaluate the safety of magnet application in patients with ICDs having Mohs or cutaneous surgery. Our surgical database was reviewed to identify all patients with an ICD who had a surgical procedure between 2017 and 2023. Details regarding their device, their surgical procedure including type of electrosurgery used and input from the electrophysiology team were recorded. The electrophysiology team was contacted for all patients in advance: they would attend and deactivate the ICD if the patient was pacing dependent or had other high risk factors for example, cardiomyopathy. Medical records were reviewed to identify any immediate recorded adverse events and clinical notes were reviewed for 3 months following procedure to note any hospital visit. The device reports were interrogated for any arrhythmias on the day of the procedure and for 4 weeks following procedure.

In the magnet cohort, a standard donut magnet 77 mm × 14 mm with a 30‐mm hole was placed directly over the device by the dermatology team, with 2‐inch tape in an X fashion before surgery. This was removed as soon as surgery was complete. For Mohs cases, the magnet was removed between surgical stages. Patients were monitored with finger pulse oximetry and an external defibrillator was on standby.

In the high risk cohort, the ICD was deactivated by the electrophysiology team, who remained present during the procedure and reactivated the device at surgery completion. This group had continuous ECG monitoring during procedure and was connected to an external defibrillator. In all procedures bipolar coagulation forceps were used, with minimal bursts and power.

We identified 18 patients with a total of 33 procedures. 20 procedures were with Cardiac Resynchronisation Therapy with Defibrillators CRT‐Ds and 13 procedures with standard ICDs. There were 6 females and 12 male patients. The median age was 79 years. The procedures comprised of 8 cases of Mohs Micrographic surgery, 17 excisions, 1 shave biopsy, 4 shave excisions, 2 Curettage & Cautery procedures, 1 incisional biopsy. Most procedures were on the head and neck and all were above the umbilicus apart from 2 procedures on the calf. The location of the lesions and diagnosis is listed in Table 1.

**TABLE 1:** List of procedures in patients with ICDs included in the study.

Gender	Device	Company	Model	Magnet application	Tumour location	Histology	Procedure	Anticoagulation
F	CRT‐D	Medtronic	Compia MRI	Yes	Left cheek	AK	Shave biopsy	Edoxaban
M	CRT‐D	Boston	Autogen	Yes	Left temple	SCC	Mohs	Warfarin
M	CRT‐D	Boston	Autogen	Yes	Right temple	SCC	Mohs	Warfarin
M	CRT‐D	Boston	Autogen	Yes	Left cheek	SCC	Mohs	Warfarin
M	CRT‐D	Boston	Autogen	Yes	Right helix	SCC	Mohs	Warfarin
M	CRT‐D	Boston	Autogen	Yes	Left shoulder	SCC	Excision	Warfarin
M	CRT‐D	Boston	Autogen	Yes	Right forearm	SCC	Excision	Warfarin
F	ICD	St Jude	Ellipse DR	No *deactivation	Right cheek	BCC	Mohs	Aspirin
F	ICD	St Jude	Ellipse DR	No *deactivation	Right temple	BCC	Mohs	Aspirin
F	ICD	St Jude	Ellipse DR	No *deactivation	Right lower eyelid	BCC	Mohs	Aspirin
M	ICD	Boston	Autogen DR	Yes	Right upper back	Dysplastic naevus	Excision	Edoxaban
M	CRT‐D	St Jude	Unify assura CRTD	Yes	Right upper chest	Dysplastic naevus	Excision	Rivaroxaban, aspirin
M	ICD	St Jude	Ellipse DR	Yes	Right nasal ala	BCC	Mohs	Warfarin
M	ICD	Biotronic	Rivacor	No *deactivation	Left helical rim	SCC	Excision	Aspirin
F	ICD	Medtronic	Visia AF MRI	Yes	Left jawline	SCC	Excision	Aspirin
F	ICD	Boston	Resonate EL	Yes	Right eyelid	BCC	Shave excision	Warfarin
M	ICD	St Jude	Fortify VR	Yes	Right axilla	Sebaceous adenoma	Excision	Warfarin, aspirin
M	ICD	St Jude	Fortify VR	Yes	Right cheek	SCC in situ	Incisional biopsy	Warfarin, aspirin
M	CRT‐D	St Jude	Unify assura	Yes	Right heliex	Actinic keratosis	C&C	Aspirin
M	ICD	Boston	Boston Momentum	Yes	Right upper back	BCC	Excision	Aspirin
F	CRT‐D	Medtronic	Cobalt HF	No *deactivation	Right cheek	SCC	Excision	Aspirin
M	ICD	Medtronic	Visis VR	Yes	Frontal scalp	AK	C&C	Warfarin
M	ICD	Boston	Teligen VR	Yes	Right cheek	SCC	Excision	Aspirin
M	CRT‐D	Medtronic	Claria MRI Quad	No *deactivation	Right chest	Junctional naevus	Excision	Rivaroxaban
F	CRT‐D	St Jude	Quadra assura MP	No *deactivation	Right chest	SCC	Excision	Aspirin
F	CRT‐D	St Jude	Quadra assura MP	No *deactivation	Left forearm	SCC	Shave excision	Aspirin
F	CRT‐D	St Jude	Quadra assura MP	No *deactivation	Left forearm	Bowen's disease	Shave excision	Aspirin
F	CRT‐D	St Jude	Quadra assura MP	No *deactivation	Left calf	SCC	Shave excision	Aspirin
F	CRT‐D	St Jude	Quadra assura MP	Yes	Left forearm	Bowen's disease	Excision	Aspirin
F	CRT‐D	St Jude	Quadra assura MP	Yes	Left forearm	Scar	Excision	Aspirin
F	CRT‐D	St Jude	Quadra assura MP	Yes	Left calf	Scar	Excision	Aspirin
M	CRT‐D	Medtronic	Amplia DTMB2QQ	No *deactivation	Right arm	BCC	Excision	Edoxaban
M	CRT‐D	Medtronic	Amplia DTMB2QQ	No *deactivation	Forehead	BCC	Excision	Edoxaban

In 21/33 procedures a magnet was placed over the cardiac device by the dermatology team. In 12 procedures electrophysiologists managed the deactivation and reactivation of the device. The reason for electrophysiology attendance was pacing dependence and in one case surgeon's choice.

Post procedure interrogation showed no detection of EMI or tachyarrhythmic events in any of the 33 procedures. Devices were fully interrogated and data gathered including battery status, lead parameters and a current arrhythmia log. No patients had clinical complications at the time of surgery or in the post operative period.

Our study demonstrates that dermatological surgery can be performed safely on patients with ICDs, without the need for high level resources or monitoring, if patients are correctly selected and managed.

Previous concerns regarding ICDs relate to EMI affecting the function of the device. Despite newer ICDs having better shielding,^3^ ICDs may still interpret EMI as a tachyarrhythmia resulting in an unwanted shock to the patient.^4^ Published studies vary widely in their preoperative, intraoperative and postoperative management of these patients.

For example, Amin et al^1^ reported no complications in their 45 patients (26 pacemakers/19 ICDs) who had monopolar diathermy, with none of the patients having a preoperative or intraoperative cardiac evaluation. Their device interrogation did not find any issues.

In contrast to this, El‐Gamal et al,^4^ in their study of reports by 166 Mohs surgeons, showed a variety of complications including asystole, firing of an ICD, skipped beats although it is not clear exactly which cases involved ICDs and which involved pacemakers. 16% of Mohs surgeons in this study routinely deactivated ICDs.

Our study is unique in its focus on ICDs and assessing the safe use of magnets applied by the dermatology team. Whist the British Society for Dermatologial Surgery (BSDS) and British Heart Rhythm Society (BHRS) have released revised guidance supporting this method,^5^ our study is the first we know of that demonstrates their safety in dermatological surgery.

We believe our data supports their use in routine clinical practice, with the time and convenience benefits to the patient and sparing the use of electrophysiology resources.

## Data Availability

The data underlying this article will be shared on reasonable request to the corresponding author.
